# Gap Junctions between Endothelial Cells Are Disrupted by Circulating Extracellular Vesicles from Sickle Cell Patients with Acute Chest Syndrome

**DOI:** 10.3390/ijms21238884

**Published:** 2020-11-24

**Authors:** Joanna Gemel, Yifan Mao, Gabrielle Lapping-Carr, Eric C. Beyer

**Affiliations:** Department of Pediatrics, University of Chicago, Chicago, IL 60637, USA; jgemel@peds.bsd.uchicago.edu (J.G.); ymao9898@gmail.com (Y.M.); glappingcarr@peds.bsd.uchicago.edu (G.L.-C.)

**Keywords:** Connexin43, endothelium, gap junction, sickle cell disease, tight junction, extracellular vesicle, endothelial integrity

## Abstract

Intercellular junctions maintain the integrity of the endothelium. We previously found that the adherens and tight junctions between endothelial cells are disrupted by plasma extracellular vesicles from patients with sickle cell disease (especially those with Acute Chest Syndrome). In the current study, we evaluated the effects of these vesicles on endothelial gap junctions. The vesicles from sickle cell patients (isolated during episodes of Acute Chest Syndrome) disrupted gap junction structures earlier and more severely than the other classes of intercellular junctions (as detected by immunofluorescence). These vesicles were much more potent than those isolated at baseline from the same subject. The treatment of endothelial cells with these vesicles led to reduced levels of connexin43 mRNA and protein. These vesicles severely reduced intercellular communication (transfer of microinjected Neurobiotin). Our data suggest a hierarchy of progressive disruption of different intercellular connections between endothelial cells by circulating extracellular vesicles that may contribute to the pathophysiology of the endothelial disturbances in sickle cell disease.

## 1. Introduction

Maintaining the integrity of the endothelial lining of blood vessels is critical to sustaining healthy tissues. Disruption of the endothelial barrier is a significant component of the pathophysiology of many diseases because it allows extravasation of normal blood contents, including fluid, proteins, and cells, as well as abnormal ones, such as tumor cells and microorganisms [1]. Endothelial cells are linked by several different classes of intercellular junctions including adherens, tight, and gap junctions. The adherens and tight junctions have been most extensively studied in relation to endothelial barrier function. The adherens junctions provide a mechanical linkage between adjacent cells, and VE-cadherin is among their most important components [2]. Tight junctions seal the extracellular space between endothelial cells and regulate the movement of water and ions through the paracellular pathway. They contain transmembrane proteins (including occludin and claudins) and cytoplasmic proteins (such as zonula occludens-1, ZO-1) [3,4]. Gap junctional channels (which allow the intercellular exchange of ions and small molecules) have been less studied, but they also appear to have important roles in maintaining vascular integrity [5,6,7,8]. Gap junctions are formed by members of a family of subunit proteins called connexins; although several different connexins (including Cx37, Cx40, and Cx43) are expressed in different endothelial cells from different vascular beds, the present study focused only on Cx43 because it was the major connexin in our cultures of microvascular endothelial cells. 

Endothelial damage and activation are central components of many of the complications of sickle cell disease (SCD) [9]. SCD is an inherited hemoglobinopathy, caused by a single amino acid substitution in the hemoglobin β-chain (Glu6→Val). This change makes the hemoglobin susceptible to polymerization, which causes a deformation of red blood cells (sickling). Many of the complications of SCD result from sickled cells adhering to the endothelium and obstructing blood flow through small vessels. Similar pathologic events contribute to damage in many different organs of the body. We have concentrated our efforts on studies of patients with the pulmonary complication, Acute Chest Syndrome (ACS).

In recent years, it has been shown that the plasma contains extracellular vesicles (EVs). EVs are small vesicles containing cellular contents, surrounded by lipid bilayers, and produced by many different kinds of cells. Circulating EVs have been proposed to modulate endothelial responses to damage [10,11]. EVs that encounter endothelial cells can affect their behavior through interactions at the cell surface or by the transfer of their contents (including proteins, lipids, DNA, mRNA, and microRNAs) that carry signals from their cell of origin. Plasma EVs may contribute to various vascular diseases (including SCD) by influencing key pathophysiologic components: Endothelial dysfunction and damage, ischemia-reperfusion injury, thrombosis, and inflammation (reviewed by [12,13]). 

We have focused our recent studies on the small EVs (50–200 nm), which are often referred to as exosomes. The small EVs have recently been implicated in many different kinds of intercellular signaling events because they contain nucleic acids (including mRNAs and microRNAs), as well as proteins and lipids. We have shown that the plasma of children and young adults with SCD contains abundant small EVs [14,15]. These EVs have the size of exosomes (~100 nm diameter) and express characteristic exosomal marker proteins (CD63 and flotillin-1) [16]. Circulating small EVs are more abundant in subjects with SCD than in control subjects [15,16]. The SCD EVs cause disruption of intact monolayers of cultured endothelial cells, as detected by reduced impedance, opening of spaces between cells, and alteration of VE-cadherin-containing adherens junctions [14,15,16]. The effects of EVs were much more severe when obtained during an episode of ACS than from the same patient at baseline [16]. 

The current study was designed to examine the effects of small EVs from subjects with SCD on endothelial cell gap junctions and intercellular communication, and to investigate the effects of EVs isolated at baseline compared to during an episode of ACS.

## 2. Results

### 2.1. Properties of Circulating EVs from Subjects with SCD 

We isolated small EVs from the plasma of control subjects and subjects with SCD. We have previously shown that these vesicles contain CD63 and flotillin-1, but do not contain resident proteins from the endoplasmic reticulum or lipoproteins [16]. We examined several samples by nanoparticle tracking analysis and by transmission electron microscopy (Supplemental Figure S1). Both techniques showed that the preparations contained relatively homogeneous populations of vesicles. Nanoparticle tracking analysis showed that the mode diameter of the particles was ~110 nm. The particles appeared smaller in the electron micrographs likely due to shrinkage during fixation and processing [17,18]. 

### 2.2. EVs Isolated during an Episode of ACS Reduce the Abundance of Cx43 (But Not Other Junctional Proteins) at Appositional Membranes

**Figure 1:**
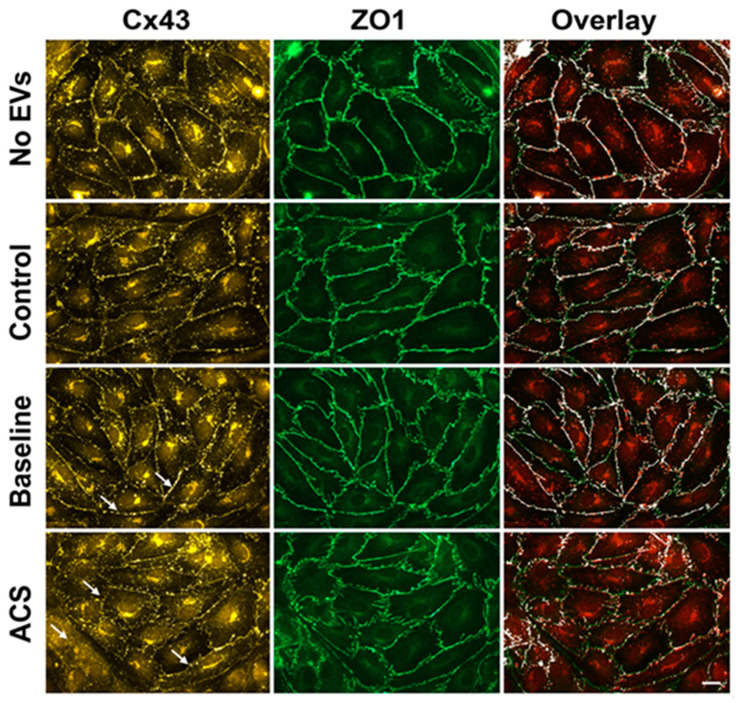
Localization of Cx43, ZO-1, and overlay in endothelial cells treated with EVs for 24 h. Representative photomicrographs are shown for HMVEC-D cells following 24 h treatment with no EVs, EVs from a control subject, EVs from a subject with SCD at baseline, and EVs from the same subject at the beginning of an episode of ACS. Cx43 (left panels, yellow/red) and ZO-1 (middle panels, green) were detected by immunofluorescence. The right panels show an overlay of the Cx43 and ZO-1 images (created using Image J), to demonstrate the co-localization of Cx43 and ZO-1 (co-localized points are shown in white). The amount of co-localization at the membrane decreased in the cells treated with EVs from an ACS sample. Arrows in the left panels point to the Cx43 staining at the membrane which was frequently, but not uniformly less abundant and continuous in cells treated with EVs from the subject with SCD during an episode of ACS, when compared with cells treated with EVs from the same subject at baseline. Scale bar is 20 µm.

We have previously observed that EVs from subjects with SCD cause a significant reduction in the physiological integrity of endothelial monolayers within 24 h, based on electric cell-substrate impedance sensing [15]. However, morphologically observable monolayer disruption (the opening of spaces between cells) requires ~48 h. In order to check for the presence of subtle changes of Cx43 and ZO-1, we initially studied cells 24 h after treatment with EVs. Representative examples of these experiments are shown in Figure 1. We compared the appearance of cells and the distributions of intercellular junction proteins in four groups of endothelial cells: Cells treated with no EVs, with EVs from controls, with EVs from subjects with SCD at baseline, or with EVs isolated from SCD subjects with SCD during an episode of ACS. None of the treatments led to significant openings of spaces between cells by 24 h. Cells treated with no EVs, with EVs from control subjects, and with EVs from subjects with SCD at baseline looked similar (Figure 1, top three rows of panels). Immunoreactive Cx43 was abundantly localized with a punctate distribution along cell membranes at points of contact between cells and in a perinuclear distribution within the cytoplasm. ZO-1 showed a continuous distribution along appositional membranes. There was extensive co-localization of Cx43 and ZO-1 (Figure 1, right panels). Cells treated with EVs isolated from subjects during an episode of ACS looked somewhat different. In many cells, the Cx43 at the membrane appeared less intense. While Cx43 immunoreactivity at appositional membranes was still punctate, it frequently showed discontinuities (arrows in Figure 1, bottom left panel). ZO-1 localization did not appear to be substantially affected by the ACS EVs. For the examples shown in Figure 1, the fraction of the ZO-1 staining that overlapped with Cx43 staining decreased from 46% for the baseline sample to 25% for the sample treated with ACS EVs. In parallel experiments, the distributions of VE-cadherin (like ZO-1) did not appear different among the different treatments for 24 h.

**Figure 2:**
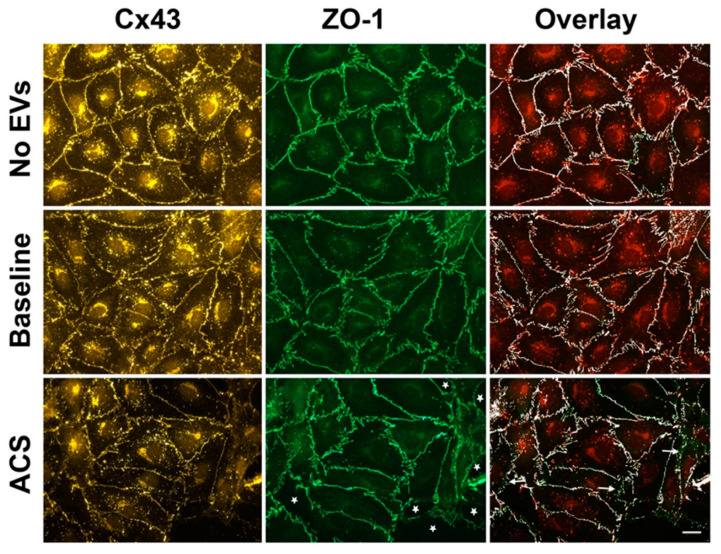
Localization of ZO-1, Cx43, and overlay in endothelial cells treated with EVs for 48 h. Representative photomicrographs are shown for HMVEC-D cells following 48 h treatment with no EVs, EVs from a subject with SCD at baseline, and EVs from the same subject at the beginning of an episode of ACS. Cx43 (left panels) and ZO-1 (middle panels) were detected by immunofluorescence. Right panels represent co-localization of Cx43 and ZO-1 (colocalized points are shown in white). Arrows indicate the regions along the membrane interfaces with poor overlay between Cx43 and ZO-1; green staining alone. The amount of co-localization at the membrane decreased in the cells treated with EVs from an ACS sample (18% less Cx43 immunoreactivity in areas that stained for ZO-1). White stars in the middle bottom panel indicate extracellular space in the ACS sample, showing damage to endothelial integrity caused by EVs from ACS patients. In the example shown, the monolayer disruption was 6.7%. Scale bar is 20 µm.

The treatment of endothelial cells with EVs from subjects with SCD for 48 h caused further changes. Representative examples of this study are shown in Figure 2. We compared cells treated with no EVs to cells treated with EVs from subjects with SCD at baseline, or during an episode of ACS (cells treated with EVs from control subjects were indistinguishable from cells treated with no EVs). The endothelial cells treated with EVs from subjects with SCD at baseline appeared rather similar to cells treated with no EVs (or control EVs): ZO-1 immunoreactivity was continuous along membranes and had similar intensities. While the intensity of Cx43 immunoreactivity was not affected by the baseline SCD EVs, it was less uniformly continuous. In contrast, the endothelial cells treated with EVs isolated from subjects during an episode of ACS looked markedly different. There was significant monolayer disruption visualized by the opening of spaces between cells (stars in Figure 2, bottom middle panel). The continuity and intensity of ZO-1 immunoreactivity was not affected at positions where cell interfaces were intact. In contrast, Cx43 staining at appositional membranes appeared less bright with many discontinuities, and occasionally was nearly absent (compare left panels in Figure 2). Consequently, in the overlay between Cx43 and ZO-1 immunostaining, there was less co-localization and more regions along the membrane interfaces that only stained for ZO-1 (arrows indicating green staining alone in Figure 2, bottom right panel).

**Figure 3:**
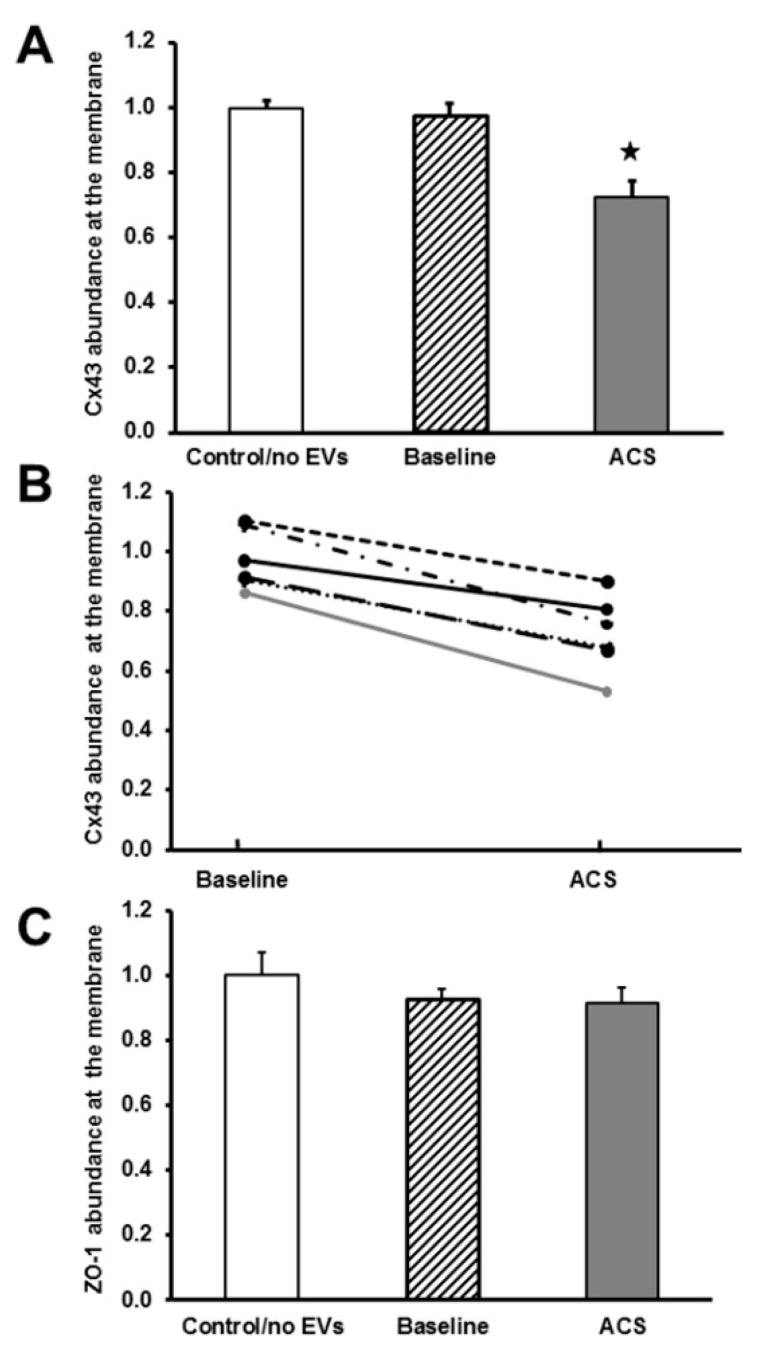
Comparison of membrane staining of Cx43 and ZO-1 in endothelial cells after treatment with ACS plasma-derived EVs. Endothelial cells were grown to confluence and then treated for 48 h with no EVs (*n* = 1), EVs from controls (*n* = 2), EVs from subjects with SCD at baseline (*n* = 6), or EVs from the same subjects during an ACS episode (*n* = 6). (**A**) Graph shows intensity of Cx43 at the membrane normalized to the average values in control/no EVs treated cells (*n* = 3). There was a dramatic reduction of Cx43 intensity at the membrane in endothelial cells treated with EVs from ACS samples (★, *p* < 0.05 as compared to baseline EV treated cells). ANOVA, followed by a Tukey post-hoc test, showed significant differences between control/no EVs and ACS as well as between baseline and ACS, (★, *p* < 0.05). Control/no EVs and baseline were not significantly different (*p* > 0.05). (**B**) Graph shows intensity of Cx43 staining at the membrane in individual subjects at baseline or during ACS (with results from the same subject connected by lines, referring to baseline and ACS episode as a pair of results for the same subject). 6 different forms of lines are used to distinguish between 6 different subjects. In all 6 subjects, EVs isolated during ACS decreased the Cx43 intensity at the membrane compared to EVs isolated during baseline. (**C**) Graph shows intensity of ZO-1 at the membrane normalized to the average values in control/no EVs. None of these values were significantly different.

We quantified the abundance of immunoreactivity for Cx43 and ZO-1 in cells, treated with each patient sample (Figure 3), as described in the Methods section and exemplified in Supplemental Figure S2. This analysis showed that there were no significant differences among samples treated with no EVs or with EVs obtained from control subjects. Therefore, these samples were considered together. We also found no significant differences in the membrane abundance of Cx43 between cells treated with baseline EVs and these controls (Figure 3A). However, there was a significant decrease (by ~30%) in the abundance of Cx43 membrane staining in cells treated with EVs, obtained during ACS episodes, compared with cells treated with EVs from subjects at baseline or controls (Figure 3A). Moreover, when we compared the abundance of Cx43, it decreased between baseline and ACS within each individual subject (Figure 3B). There were no significant differences in the intensity of ZO-1 membrane staining among endothelial cells treated with the different EVs (Figure 3C).

**Figure 4:**
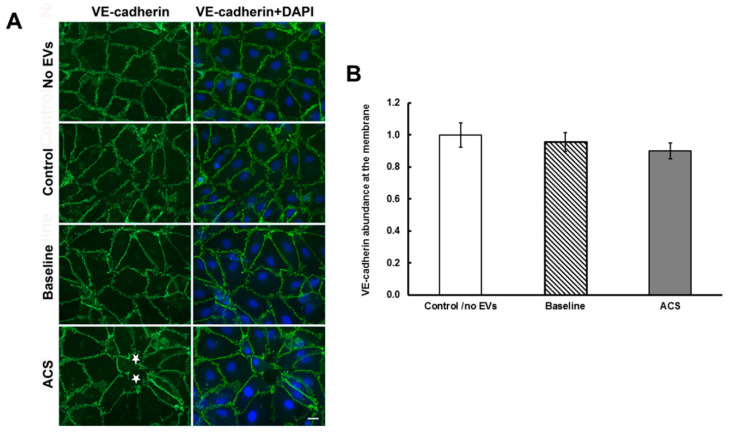
Localization of VE-cadherin and nuclei in endothelial cells treated with EVs. (**A**) Representative photomicrographs are shown for HMVEC-D cells 48 h after treatment with no EVs, EVs from a control subject, EVs from a subject with SCD at baseline, and EVs from the same subject at the beginning of an episode of ACS. VE-cadherin was detected by immunofluorescence (green) and nuclei by staining with DAPI (blue). In the example shown in the bottom row for an ACS sample, the monolayer disruption was 1.4%. White stars indicate spaces between cells. Scale bar is 20 µm. (**B**) The extent of VE-cadherin at the membrane (normalized integrated intensity) was calculated using Image J software. No significant differences were found (using the same approach as for the analysis in Figure 3). Control/no EVs *n* = 3; baseline and ACS *n* = 5.

We similarly examined VE-cadherin and quantified its abundance at the plasma membrane in endothelial cells treated with no EVs, with EVs from control subjects, or with EVs from subjects with SCD at baseline or during an episode of ACS (Figure 4). The ACS EVs led to the opening of spaces between some cells (stars in Figure 4A, bottom left panel) and loss of immunoreactive VE-cadherin from the adjacent free edges of cells. Otherwise, there was little difference among cells receiving the different treatments. Although, there appeared to be a slight downward trend, there were no significant differences in the abundances of VE-cadherin at appositional plasma membranes among cells treated with control, baseline, or ACS EVs (Figure 4B).

### 2.3. EVs Isolated during an Episode of ACS Cause Decreases in Cx43 mRNA and Protein Levels

**Figure 5:**
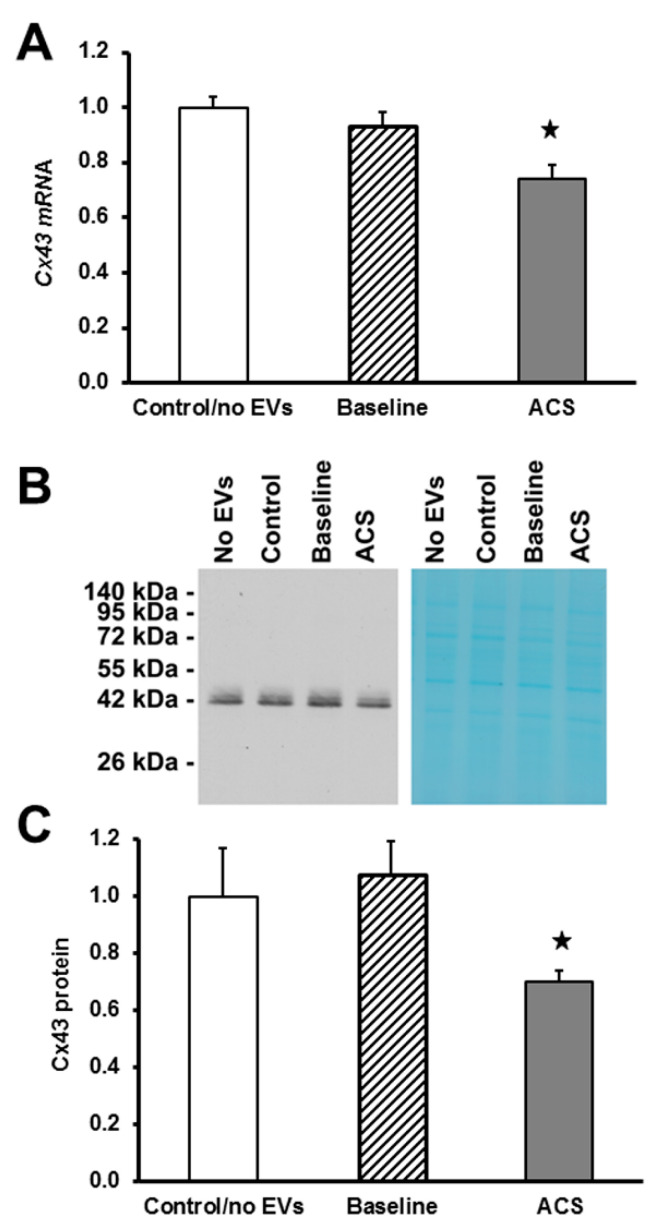
Levels of endothelial Cx43 after treatment with ACS plasma-derived EVs. (**A**) RNA was isolated and Cx43 mRNA levels were quantified by real time RT-qPCR. The graphs show the values normalized to the average values in control/no EVs (*n* = 3) treated cells. There was a significant decrease in Cx43 mRNA levels in cells treated with ACS EVs (*n* = 8) (★, *p* < 0.05 as compared to baseline EVs treated cells by ANOVA followed by Tukey post-hoc test). (**B**) Representative blot for Cx43 is shown (left panel). MEM Code Reversible Protein Staining of PVDF membrane proves loading of equal amounts of total protein in each lane (right panel). (**C**) Cx43 protein levels were quantified by densitometry. Graphs show the amounts of immunoreactive connexin in each group, normalized to the control/no EVs treated cells. Cx43 protein levels were significantly decreased in cells treated with ACS EVs, compared to cells treated with baseline EVs or control/no EVs (★, *p* < 0.05 ANOVA followed by Tukey post-hoc test).

To further explore the disruption of gap junctions by EVs from SCD subjects, we examined the RNA and protein levels for Cx43 in homogenates, prepared from HMVEC-D cells after treatment for 48 h with EVs. Cx43 mRNA levels did not differ between endothelial cells treated with control/no EVs or EVs obtained from subjects at baseline (Figure 5A). However, endothelial Cx43 mRNA levels were significantly decreased (by ~25%) in cells treated with EVs obtained from patients during an ACS episode (Figure 5A). Consistently, immunoblots revealed that Cx43 levels were not different between endothelial cells treated with control/no EVs or EVs obtained from subjects at baseline, but they were decreased (by ~30%) in cells treated with ACS EVs (Figure 5B,C).

### 2.4. ACS EVs Reduce the Extent of Gap Junction-Mediated Intercellular Communication

**Figure 6:**
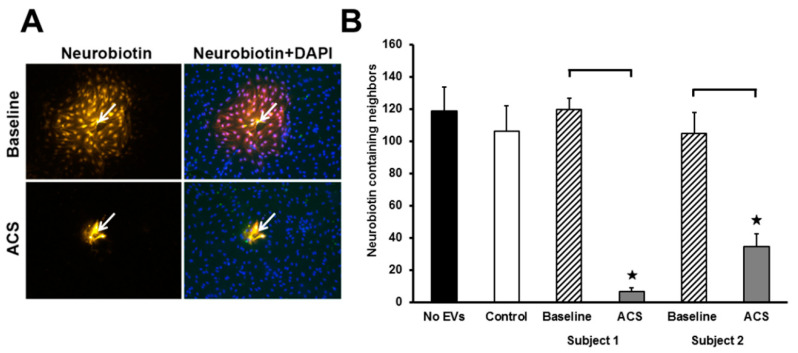
Effect of ACS plasma-derived EVs on the extent of gap junction-mediated intercellular communication. (**A**) Representative photomicrographs of intercellular transfer of Neurobiotin after its injection into a single cell (indicated by arrows) within a monolayer of endothelial cells, treated for 48 h with EVs, isolated at baseline and during an episode of ACS. The Neurobiotin tracer was detected after staining the cells with streptavidin-Cy3 conjugate (yellow). Nuclei were stained with DAPI (blue) to show the locations of all cells. (**B**) Graph shows the quantitation of Neurobiotin transfer experiments (numbers of tracer-containing neighbors) (*n* = 6–9 injections for each condition). The ACS EVs caused decreased Neurobiotin transfer to neighboring cells (decreased intercellular communication) (★ = paired *t*-test comparing ACS and baseline within SCD subjects, *p* < 0.05).

To test the extent of gap junction-mediated intercellular communication, individual cells were microinjected with the gap junction tracer Neurobiotin, which should transfer extensively to neighboring (healthy/untreated) endothelial cells. Endothelial cells treated for 48 h with EVs isolated from subjects with SCD at baseline showed extensive intercellular transfer of Neurobiotin, while cells treated with EVs isolated during an episode of ACS showed little Neurobiotin transfer (Figure 6A).

Intercellular communication was quantified by counting the number of cells adjacent to the microinjected cell that contained the Neurobiotin tracer. Endothelial cells treated with no EVs, cells treated with EVs from a control subject, and cells treated with EVs from two subjects with SCD at baseline showed extensive intercellular transfer of Neurobiotin to more than 100 coupled cells (Figure 6B). In contrast, treatment for 48 h with EVs isolated from two different subjects with SCD during ACS episodes decreased the extent of Neurobiotin transfer by 94%, and 67%, respectively.

## 3. Discussion

In this study, we have shown that small EVs isolated from the plasma of human subjects with SCD (collected during an episode of ACS) cause the disruption of gap junctions between cultured endothelial cells. EVs obtained during an episode of ACS had consistently greater effects than ones isolated at baseline from the same subject. The EVs caused reductions of Cx43 mRNA, protein, and immunoreactivity at appositional membranes (gap junction plaques), as well as a reduction of intercellular communication (transfer of Neurobiotin). The reductions in Cx43 mRNA and protein likely reflect some decrease in synthesis. The loss of Cx43 gap junctions and reductions of protein levels and cell-cell communication may also be due to degradation of this junctional protein that has a half-life of only a few hours [19,20]. The greater reduction of dye transfer than Cx43 protein levels detected by immunoblotting or immunostaining suggest that there may also have been a reduction of Cx43 channel function. 

**Figure 7:**
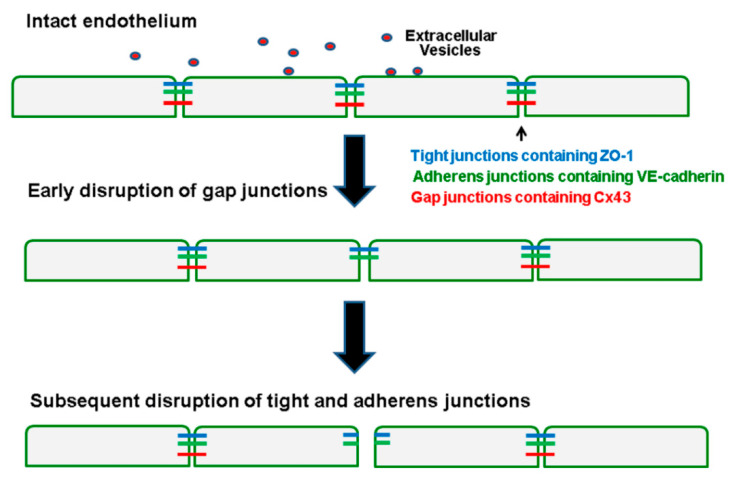
Model illustrating the progressive sequence of changes in the endothelium caused by EVs isolated from a subject with SCD during an ACS episode. After the endothelial monolayer is treated with EVs, there is early disruption of gap junctions containing Cx43 (red), followed by subsequent disruption of tight junctions containing ZO-1 (blue) and adherens junctions containing VE-cadherin (green).

This work extends our previous studies demonstrating the deleterious effects of SCD EVs on endothelial cell tight junctions and adherens junctions [15,16]. In the current study, the gap junctions appeared to be more sensitive to disruption by the EVs and to be affected earlier (detectable effects at 24 h) than the other types of intercellular junctions. These findings lead us to propose the model shown in Figure 7 illustrating the sequence of events in endothelial cells caused by EVs from subjects with ACS. Circulating EVs from the individuals with SCD (and ACS) encounter endothelial cells and transfer a “disruptive signal” (possibly by delivery of microRNAs). The first detectable junction-related event is some loss of Cx43-containing gap junctions. At later times (48 h), gap junctions are further reduced, while tight and adherens junctions are also disrupted. Hierarchical or progressive changes in interactions between connexins and proteins from other classes of junctions have been previously observed [21,22,23,24]. There are several possible explanations for this temporal sequence. It is possible that a reduction of gap junctions, connecting endothelial cells decreases the intercellular passage of a signaling molecule that prompts formerly coupled cells to degrade other kinds of intercellular junctions. Connexins are linked to some cytoskeletal components that also interact with other junctional molecules [25,26]; it is possible that loss of connexins destabilizes this complex. Reductions of other types of junctional molecules after Cx43 might be due to decreased transcription/translation of those molecules that is directly linked to Cx43 [27] or they may be independent effects of the EVs. A previous study also concluded that changes in Cx43-containing gap junctions altered other endothelial junctions [28]. However, in that case, increased Cx43 was associated with increased permeability of tight junctions in cerebral cavernous malformations. 

As small EVs contain microRNAs, and because the effects that we observed took days (24–48 h), it is tempting to suggest that microRNAs from the EVs entered the target endothelial cells and led to gene expression changes for the different junction proteins. There have been a number of previous papers showing that microRNAs affect the expression of adherens and tight junction proteins in endothelial cells [29,30,31,32,33,34]. Although, not extensively studied in endothelial cells, there is strong evidence that several microRNAs (including miR-1, miR-130a, miR-206, and others) can downregulate Cx43 [35,36,37]. 

The current observations extend our understanding of the repertoire of roles of gap junctions between endothelial cells. Gap junction-mediated intercellular communication in the endothelium has been known for many years [6]. Communication between these cells has been implicated in regulation of vasomotor tone [7,38]. Endothelial gap junctions have also been implicated in other vascular diseases including atherosclerosis, thrombosis, and ischemia-reperfusion injury (reviewed in [39]). There is limited information regarding connexins and endothelial barrier function [8], although several studies implicate connexins in regulating the blood-brain barrier (which is comprised of multiple cell types) [40,41]. Some connexin-mediated effects in vascular endothelial cells may result from opening of hemi-channels [42,43,44]. However, we did not study connexin hemi-channels.

We studied Cx43-containing gap junctions, because it is the most abundantly expressed connexin in cultured endothelial cells [45], including the ones that we have previously used to study the effects of EVs on tight and adherens junctions. Cx43 is present in the gap junctions connecting some endothelial cells in vivo, but Cx37 and Cx40 also form many inter-endothelial gap junctions [46,47]. Elucidation of the effects of EVs upon endothelial gap junctions composed of these other connexins (which are abundant in many vascular beds, including in the lung) must await further studies utilizing intact animals or tissues.

## 4. Materials and Methods 

### 4.1. Subjects with Sickle Cell Disease and Controls

Details of subjects with SCD, and control subjects, have been described thoroughly previously [16], including demographic and clinical characteristics. In brief, we identified 9 subjects with SCD who had plasma samples obtained both, at baseline and at the beginning of an admission for ACS, prior to transfusion. The SCD subjects were prospectively enrolled in our biobank, with informed consent provided by parents and assent obtained from subjects 9–18 years of age. The children with SCD ranged from 3–19 years of age and included 6 females and 3 males. Baseline samples were obtained from SCD subjects in a steady state of disease (free of infection, new pain, or transfusion for at least 4 weeks prior to blood sample collection). ACS was defined as a new infiltrate on chest x-ray accompanied by fever, hypoxia, tachypnea, cough, or chest pain. Control subjects (*n* = 6) were recruited from the general pediatric clinic; they included both males and females, and they were age-matched with the SCD subjects. They had a BMI < 85th percentile, did not carry a diagnosis of asthma or other inflammatory disorder, and were having blood drawn for screening or monitoring of iron deficiency. Baseline SCD and control subject blood samples were obtained at the time of a clinic visit. ACS samples were obtained from fasting subjects at an early morning blood draw during the first 24 h of a hospital admission. 

All protocols were approved by the Institutional Review Board at the University of Chicago (protocol # 14-0466 on 12/16/2014 and protocol # 15-0263 on 5/15/2015) and were conducted in accordance with the guidelines set by the Declaration of Helsinki.

### 4.2. Isolation of Small Extracellular Vesicles

EVs were isolated from platelet-free plasma using the Total Exosome Isolation kit (Thermo Fisher Scientific Inc., Waltham, MA, USA) according to the manufacturer’s guidelines, as we have done previously [14,15]. Each isolation started with 100 µL of plasma. EV pellets were resuspended in a final volume of 50 µL of phosphate buffered saline, pH 7.4 (PBS). Nanoparticle tracking analysis was performed using the Nanosight NS300 (Malvern Panalytical Inc., Westborough, MA, USA), as we have done previously [13]. Isolated EVs were diluted with PBS (1:100–1:150) and injected into the 488 nm laser chamber with a constant output controlled by a syringe pump. Three recordings were performed for each sample. Nanoparticle tracking analysis software was used to measure nanoparticle size. Imaging of negatively stained EVs by transmission electron microscopy was performed in the Advanced Electron Microscopy Core Facility at the University of Chicago with the assistance of Dr. Tera Lavoie.

### 4.3. Primary Endothelial Cell Culture

Human dermal microvascular endothelial cells (CC-2543), HMVEC-D (purchased from Lonza Walkersville, Inc.; Allendale, NJ, USA), were maintained, according to the manufacturer’s instructions in endothelial growth medium (EGM-2MV Bullet Kit; Lonza) and incubated at 37 °C, 5% CO_2_ in a cell culture incubator. All experiments were performed at passage 10.

### 4.4. Antibodies

Cx43 was detected using rabbit polyclonal antibodies directed against amino acids 363–382 of human/rat Cx43 (C6219, SIGMA-Aldrich, St. Louis, MO, USA) at 1:5000 dilution for immunoblotting and at 1:250 dilution for immunofluorescence. Mouse monoclonal antibody against human recombinant ZO-1 fusion protein encompassing amino acids 334–634 (cat no 33-9100, Thermo Fisher Scientific Inc.) was diluted 1:250 for immunofluorescence. VE-cadherin was detected using mouse monoclonal antibodies (sc-9989, Santa Cruz, Biotechnology, Inc., Santa Cruz, CA, USA) at 1:100 dilution for immunofluorescence.

AlexaFluor 488 goat anti-mouse IgG and horseradish peroxidase (HRP)-conjugated goat anti-rabbit or anti-mouse IgG antibodies were obtained from Jackson ImmunoResearch (West Grove, PA, USA) and used according to manufacturer’s instructions.

### 4.5. Immunohistochemistry of Endothelial Cells

HMVEC-D cells were grown to confluence on glass coverslips that had been treated for 5 min with 5 µg/mL fibronectin in 0.02% gelatin (SIGMA-Aldrich, St. Louis, MO, USA) in a 12-well dish. Once confluent, cells were treated for 24–48 h with full growth media or EVs from controls or subjects with SCD diluted at 1:50 in this medium as previously described [16]. Slides were fixed with 4% paraformaldehyde in PBS for 15 min at room temperature (RT) and then washed with PBS. Cell membranes were permeabilized with 1% Triton X-100 in PBS for 10 min at RT. After washing with PBS, samples were blocked twice for 30 min each at RT with PBS containing 1% Triton X-100 and 10% normal goat serum. Cells were incubated with primary antibodies overnight at 4 °C. After three 5 min washes with PBS, cells were incubated with secondary antibodies for 1 h. For nuclear counterstaining, cover slips were incubated for 15 min with 500 µg/mL DAPI (4’,6-Diamidino-2-Phenylindole, Dihydrochloride) (Thermo Fisher Scientific Inc.) and again washed extensively with PBS. Coverslips were mounted on glass slides using Prolong Gold anti-fade reagent (Thermo Fisher Scientific Inc.). Slides were sealed and stored in darkness at 4 °C. Cells were imaged using the 10X or 40X Plan Apochromat objective in an Axioplan 2 microscope (Carl Zeiss Meditec, Munich, Germany). Images were captured with a Zeiss Axiocam digital camera using Zeiss AxioVision software. The cells were examined using the appropriate filters for AlexaFluor 488, Cy3, and DAPI. For each sample, 18 photos were taken; one photo using each filter, then in six different areas across the coverslip to gain a more complete view of all the cells on the coverslip. To avoid bias, microphotography and image analysis were performed by a team member who was not aware of the source of EVs used for the treatment of cells.

Image J 1.47v software; Bethesda, MD, USA (http://rsb.info.nih.gov/ij/) was used to analyze images. Images from all channels were overlaid to visualize all stained components. Monolayer disruption was quantified by determining the percentage of each 10X image occupied by intercellular space as detailed in [16]. Quantification of co-localization was performed by calculating the Manders’ coefficients, as we have done previously [48]. 

### 4.6. Assay of Intercellular Communication Using Micro-Injected Neurobiotin

For microinjection studies, cells were cultured on glass coverslips and treated with EVs as described above. The coverslip was transferred to F-12 medium (Thermo Fisher Scientific Inc.) buffered with 15 mM HEPES. Individual cells within clusters were impaled and injected with 5% Lucifer yellow (charge = −2, MW = 456; Thermo Fisher Scientific Inc.) and 5% Neurobiotin (charge = +1, MW = 322.8; Vector Laboratories, Burlingame, CA dissolved in water) for 1 min using a picospritzer (model PLI-188, Nikon Instruments, Melville, NY, USA). Inclusion of the Lucifer yellow tracer allowed visualization of successful microinjection of the impaled cell under UV illumination. After injection, cells were fixed in 4% paraformaldehyde for 30 min and permeabilized for 2 min with methanol/acetone (1:1) at RT. The Neurobiotin tracer was detected via staining with Cy3-streptavidin conjugate (Sigma-Aldrich) at 1:1000 dilution for 30 min at RT. Intercellular transfer of tracers was quantified by counting the number of adjacent cells containing the tracer.

### 4.7. Immunoblotting of EVs and Endothelial Cell Lysates

Cell homogenates were prepared from 60 mm dishes in PBS buffer containing 150 mM NaCl, 1% Triton X-100, 0.02% sodium azide, 50 mM sodium fluoride, 0.5 mM sodium orthovanadate, and Roche mini EDTA-free protease inhibitors (Roche Applied Science, Indianapolis, IN, USA) (one tablet per 5 mL of lysis buffer). Aliquots containing 5 μg of protein from cell homogenates were separated by SDS-PAGE on 10% gels. The protein concentrations of cell homogenates were determined using the Bradford method [49]. Proteins were blotted onto Immobilon-P membranes (Millipore, Bedford, MA, USA). ProSieve Protein Colored Markers standards (Lonza) were used to calibrate the gels. MEM Code Reversible Protein Stain Kit for PVDF membranes (Thermo Fisher Scientific Inc.) was used for staining proteins after transfer to the membranes to confirm equal loading and efficient electrotransfer. Immunoblots were developed with ECL Prime chemiluminescence reagents (GE Healthcare Biosciences, Pittsburgh, PA, USA). Subsequently, blots were exposed to X-ray film. Blots were performed for two or three independent experiments for each sample and were quantified by densitometry.

### 4.8. Isolation of RNA and Quantification of mRNA Levels

RNA was isolated from HMVEC-D cells (grown in 6-well tissue culture plates) using the miRNeasy Mini Kit (Qiagen, Germantown, MD, USA). The quality of RNA was assessed using an Agilent 2100 Bioanalyzer (Agilent Technologies, Inc., Santa Clara, CA, USA). All samples had RNA Integrity Number (RIN) values in the excellent quality range (7.7–10.0). The levels of mRNA were quantified using real-time quantitative PCR (RT-qPCR), as previously described [33]. cDNA was prepared from total RNA (1 μg) using oligo-dT and random primers with QuantiTect Reverse Transcription Kit (Qiagen). RT-qPCR analysis was performed using SYBR green (Thermo Fisher Scientific Inc.) in 96-well plates in a 7500 FAST Applied Biosystems instrument. All reactions were run in triplicate, amplified at 95 °C for 20 s, followed by 40 cycles of 95 °C for 3 s, and 60 °C for 30 s. The primer sequences for amplification of Cx43 matched those that we have used previously [48]; they were synthesized by Integrated DNA Technologies, Inc., Coralville, Iowa. After testing the human housekeeping gene primer set (HHK-1; Real Time Primers, LLC, Elkins Park, PA, USA), human ribosomal protein L13a, RPL13A was chosen as a housekeeping gene. Each experiment included negative control samples lacking template or reverse transcriptase. The relative expression of mRNA was calculated using the delta-delta CT method and normalized to the expression of RPL13A as previously described [50]. 

### 4.9. Statistical Analysis

All data were expressed as the mean ± standard error of the mean. Differences between two groups were compared by the Student’s *t*-test. Analysis of variance (ANOVA) with the Tukey post hoc multiple comparison test was performed when comparing more than two populations. *p* < 0.05 was considered to be statistically significant. GraphPad Prism9 software (San Diego, CA, USA) was used to perform analysis.

## 5. Conclusions 

Circulating EVs in subjects with SCD affect multiple components of endothelial junctions. Gap junctions composed of Cx43 are the most sensitive of the cell-cell junctions since their abundance and function are reduced by EVs (isolated from subjects with SCD and Acute Chest Syndrome) even when the endothelial monolayer appears intact. Cx43-mediated intercellular communication may be an early and sensitive event in the endothelial disturbance, which are caused by EVs in subjects with SCD.

## Supplementary Materials

Supplementary Materials can be found at https://www.mdpi.com/1422-0067/21/23/8884/s1. Figure S1: Characterization of small EVs isolated from the plasma of a subject with SCD. Figure S2. Illustrations and description of the method used for quantifying membrane staining utilizing Image J software.

## Abbreviations

ACS	Acute Chest Syndrome
Cx37	Connexin37
Cx40	Connexin40
Cx43	Connexin43
EVs	Extracellular vesicles
HMVEC-D	Human dermal microvascular endothelial cells
RPL13A J	Human ribosomal protein L13a
RT	Room Temperature
SCD	Sickle Cell Disease
ZO-1	Zonula occludens-1
