# Supplementary material for: Gap Junctions between Endothelial Cells Are Disrupted by Circulating Extracellular Vesicles from Sickle Cell Patients with Acute Chest Syndrome
Source: Int J Mol Sci. 2020 Nov 24;21(23):8884. doi: 10.3390/ijms21238884 (PMC7727676; doi:10.3390/ijms21238884)
Supplement: Supplementary file 1 [file ijms-21-08884-s001.zip › Supplemental Figure S1.pdf]

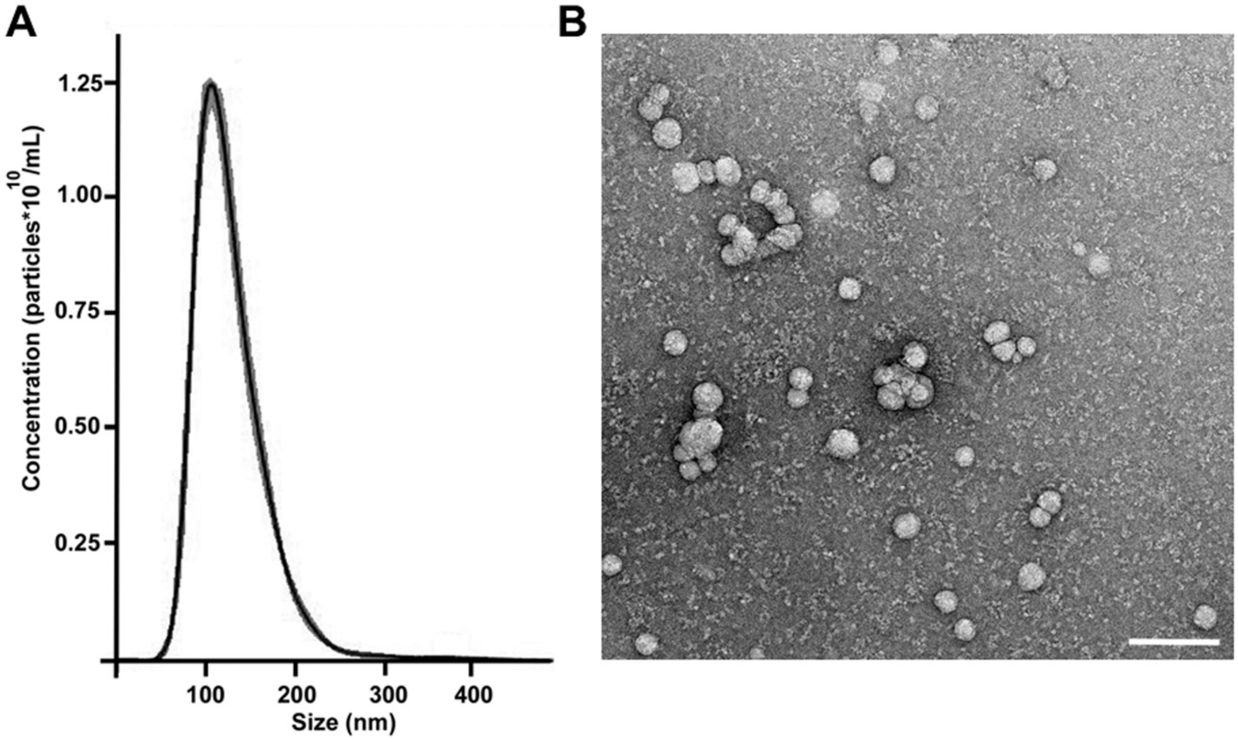


**Figure S1.** The plasma of patients with SCD contains small EVs. Small EVs were isolated from the plasma of a subject with SCD by precipitation using the Total Exosome Isolation Kit (Thermo Fisher Scientific Inc.) (A) Graph shows the distribution of particles based on nanoparticle tracking analysis. The mode diameter of EVs was 110 nm. (B) Thin section micrograph illustrates the appearance of particles after negative staining. Scale bar is 100 nm. As in other studies, the particles appear smaller by electron microscopy, perhaps due to shrinkage during specimen preparation [17, 18]. Both techniques show that the plasma of this patient contains a relatively homogenous population of small vesicles.
