# Supplementary material for: Gap Junctions between Endothelial Cells Are Disrupted by Circulating Extracellular Vesicles from Sickle Cell Patients with Acute Chest Syndrome
Source: Int J Mol Sci. 2020 Nov 24;21(23):8884. doi: 10.3390/ijms21238884 (PMC7727676; doi:10.3390/ijms21238884)
Supplement: Supplementary file 1 [file ijms-21-08884-s001.zip › Supplemental Figure S2.pdf]

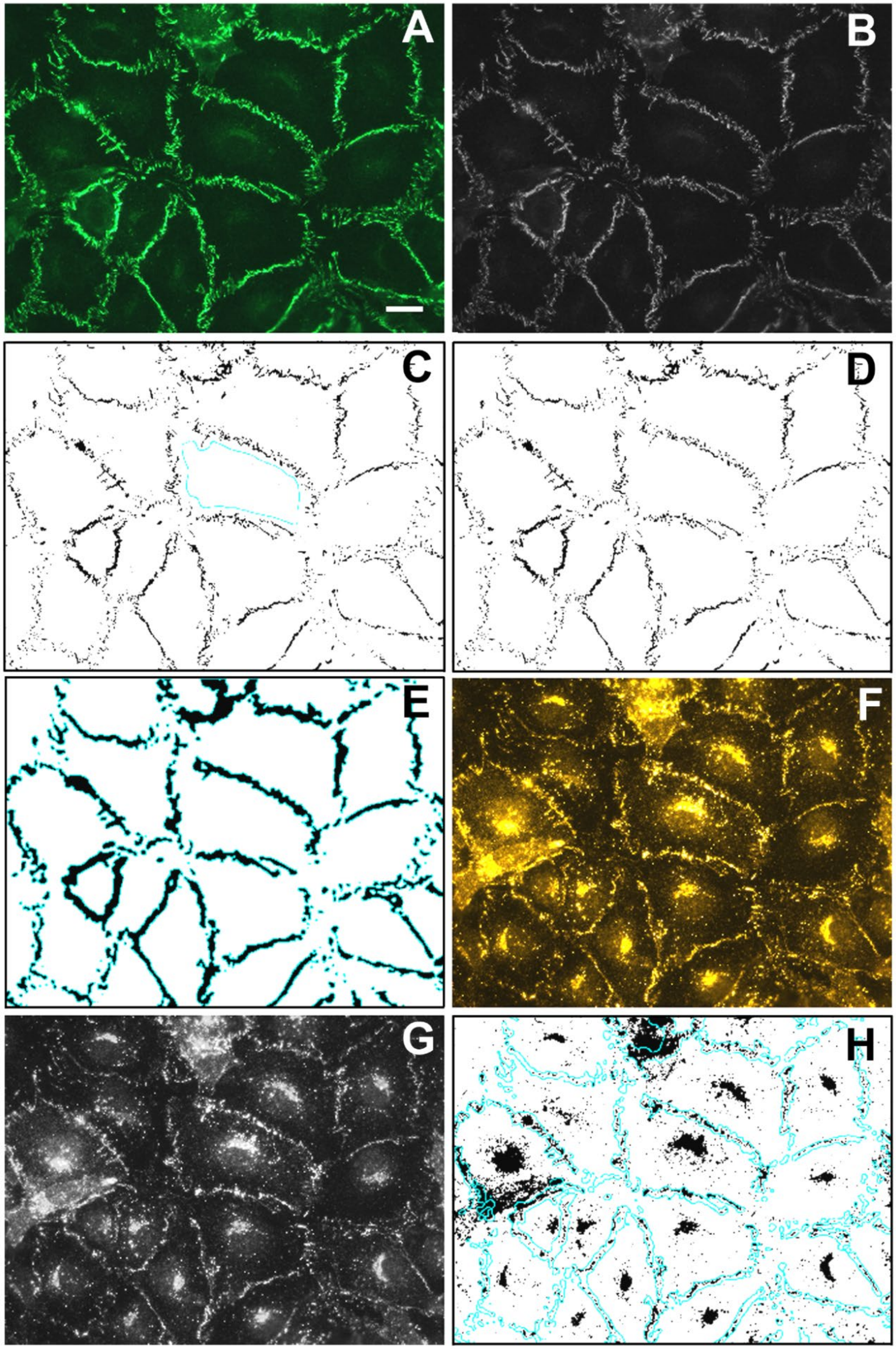
A
B
C
D
E
F
G
H

Figure S2. Example illustrating the method used for quantifying membrane staining utilizing Image J software. Images show endothelial cells studied 48 h after treatment with no EVs. (A) Photomicrograph of endothelial monolayer with immunofluorescent staining for ZO-1. This image (and all images used for quantification) was obtained using the 40X objective. Scale bar is 20 μm. (B) The micrograph was converted to a grey scale image. (C) The Image J automatic pixel intensity level threshold function was applied to show the ZO-1 membrane staining. (D) Intracellular staining was cut out from each cell using the freehand selection tool to remove any staining except at the membranes. (E) The Image J Gaussian blur filter was applied to smooth the staining at the membrane, and the membrane was selected in a cyan outline. The amount of ZO-1 staining at the membrane (integrated density) was measured using the command "Measure" in the Analyze menu. (F) Photomicrograph shows immunofluorescent staining of endothelial monolayer for Cx43. (G) The micrograph was converted to a grey scale image. (H) The Image J automatic pixel intensity level threshold function was applied to show the Cx43 membrane staining. The membrane selected from the ZO-1 photomicrograph was restored on this Cx43 photomicrograph in a cyan outline. The amount of Cx43 staining at the membrane (integrated density) was measured using the command "Measure" in the Analyze menu. To quantify the area of stained cells in each image, the amount of area without any cells (extracellular holes due to monolayer disruption) was subtracted from the total image area, after performing calculations as described in [13]. To be able to make comparisons of the intensity of staining at the membrane, integrated density for the analyzed protein (ZO-1 or Cx43) was divided by the area of cells in the analyzed image.
